# 2-{3-[1-(3,4-Dichloro­phen­yl)eth­yl]-1,3-thia­zolidin-2-yl­idene}malononitrile

**DOI:** 10.1107/S1600536810024049

**Published:** 2010-06-26

**Authors:** Xiao-jun Zhang, Hong-xin Li, Liang-zhong Xu

**Affiliations:** a College of Chemistry and Molecular Engineering, Qingdao University of Science and Technology, Qingdao 266042, People’s Republic of China

## Abstract

In the title compound, C_14_H_11_Cl_2_N_3_S, the thia­zole ring is in an envelope conformation with the –CH_2_– group bonded to the S atom forming the flap. The crystal structure is stabilized by weak inter­molecular C—H⋯Cl and C—H⋯N hydrogen bonds.

## Related literature

For the biological activity of thia­zole componds, see: Hense *et al.* (2002 ▶). For the synthesis of the title compound, see: Jeschke *et al.* (2002 ▶). For a related structure, see: Cunico, *et al.* (2007 ▶).
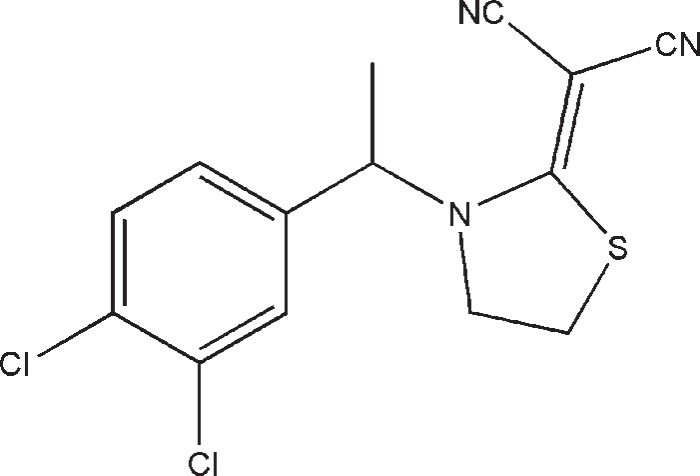


## Experimental

### 

#### Crystal data

C_14_H_11_Cl_2_N_3_S
*M* *_r_* = 324.22
Monoclinic, 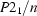

*a* = 7.5900 (15) Å
*b* = 14.957 (3) Å
*c* = 12.783 (3) Å
β = 99.03 (3)°
*V* = 1433.2 (5) Å^3^
*Z* = 4
Mo *K*α radiation
μ = 0.59 mm^−1^
*T* = 113 K
0.14 × 0.12 × 0.10 mm

#### Data collection

Rigaku Saturn diffractometer
Absorption correction: multi-scan (*CrystalClear*; Rigaku, 2005 ▶) *T*_min_ = 0.922, *T*_max_ = 0.943
8837 measured reflections
2493 independent reflections
2374 reflections with *I* > 2σ(*I*)
*R*_int_ = 0.032

#### Refinement

*R*[*F* ^2^ > 2σ(*F* ^2^)] = 0.047
*wR*(*F* ^2^) = 0.102
*S* = 1.17
2493 reflections
182 parameters
H-atom parameters constrained
Δρ_max_ = 0.98 e Å^−3^
Δρ_min_ = −0.31 e Å^−3^

### 

Data collection: *CrystalClear* (Rigaku, 2005 ▶); cell refinement: *CrystalClear*; data reduction: *CrystalClear*; program(s) used to solve structure: *SHELXS97* (Sheldrick, 2008 ▶); program(s) used to refine structure: *SHELXL97* (Sheldrick, 2008 ▶); molecular graphics: *SHELXTL* (Sheldrick, 2008 ▶); software used to prepare material for publication: *SHELXTL*.

## Supplementary Material

Crystal structure: contains datablocks I, global. DOI: 10.1107/S1600536810024049/lh5066sup1.cif

Structure factors: contains datablocks I. DOI: 10.1107/S1600536810024049/lh5066Isup2.hkl

Additional supplementary materials:  crystallographic information; 3D view; checkCIF report

## Figures and Tables

**Table 1:** Hydrogen-bond geometry (Å, °)

*D*—H⋯*A*	*D*—H	H⋯*A*	*D*⋯*A*	*D*—H⋯*A*
C3—H3*A*⋯N3^i^	0.99	2.57	3.477 (4)	153
C7—H7*A*⋯Cl2^ii^	1.00	2.83	3.623 (3)	137

Symmetry codes: (i) 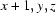; (ii) 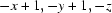.

**Fig. 1.**
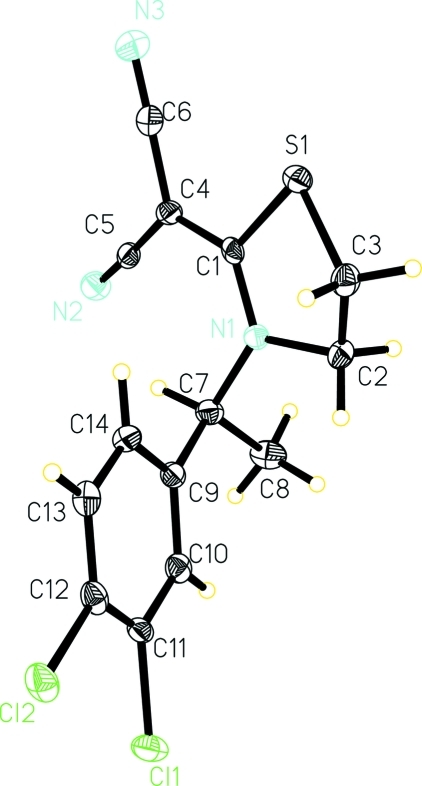
The molecular structure of (I), with displacement ellipsoids drawn at the 50% probability level.

## supplementary crystallographic information

### Comment

Recently, compounds containing the 2-(thiazolidin-2-ylidene)malononitrile group have attracted much interest because compounds containing a thiazole ring system are well known as efficient insecticides and pesticides (Hense, *et al.*, 2002). In an attempt to synthesize these types of compounds with higher biological activity the title compound (I) was synthesized and its crystal structure is reported herein.

In (I) (Fig. 1), the bond lengths angles are normal and in a agreement with those common to a previously reported structure (Cunico, *et al.*, 2007). The thiazole ring is in an envelope conformation with the -CH_2_- group bonded to the S atom forming the flap. The crystal structure is stabilized by weak intermolecular C—H···Cl and C—H···N hydrogen bonds.

### Experimental

Following the procedure of Jeschke, *et al.* (2002) 2-(thiazolidin-2-ylidene)malononitrile 15.1 g(0.10 mol) and potassium carbonate 16.6 g(0.12 mmol) were dissolved in *N*,*N*-dimethylformamide(DMF) (55 ml) and stirred 0.5 h at room temperature. Then 1,2-dichloro-4-(1-chloroethyl)benzene 20.9 g (0.10 mmol) was added, dropwise within 2 h at 318 K. The mixture was then stirred for 8 h at 358 K. After cooling at room temperature, 20 ml of water was added. The mixture was extracted with CH_2_Cl_2_ (15 ml) and the organic layer was washed with water and dried over anhydrous sodium sulfate. The excess CH_2_Cl_2_ was removed on a water vacuum pump obtaining the oily product which was rerystallized from methanol to afford the title compound 26.8 g (83% yield). Single crystals suitable for X-ray measurement were obtained by recrystallization of the title compound from a mixture of acetone and methanol at room temperature.

### Refinement

All C-bound H atoms were placed in calculated positions, with C—H = 0.95–1.00 Å, and included in the final cycles of refinement using a riding model, with *U*_iso_(H) = 1.2*U*_eq_(C).

### Crystal data

**Table:**

C_14_H_11_Cl_2_N_3_S	*F*(000) = 664
*M**_r_* = 324.22	*D*_x_ = 1.503 Mg m^−^^3^
Monoclinic, *P*2_1_/*n*	Mo *K*α radiation, λ = 0.71073 Å
Hall symbol: -P 2yn	Cell parameters from 3119 reflections
*a* = 7.5900 (15) Å	θ = 2.1–27.2°
*b* = 14.957 (3) Å	µ = 0.59 mm^−^^1^
*c* = 12.783 (3) Å	*T* = 113 K
β = 99.03 (3)°	Prism, black
*V* = 1433.2 (5) Å^3^	0.14 × 0.12 × 0.10 mm
*Z* = 4	

### Data collection

**Table:**

Rigaku Saturn diffractometer	2493 independent reflections
Radiation source: fine-focus sealed tube	2374 reflections with *I* > 2σ(*I*)
graphite	*R*_int_ = 0.032
Detector resolution: 7.31 pixels mm^-1^	θ_max_ = 25.0°, θ_min_ = 2.7°
ω scans	*h* = −9→8
Absorption correction: multi-scan (*CrystalClear*; Rigaku, 2005)	*k* = −17→17
*T*_min_ = 0.922, *T*_max_ = 0.943	*l* = −15→13
8837 measured reflections	

### Refinement

**Table:**

Refinement on *F*^2^	Primary atom site location: structure-invariant direct methods
Least-squares matrix: full	Secondary atom site location: difference Fourier map
*R*[*F*^2^ > 2σ(*F*^2^)] = 0.047	Hydrogen site location: inferred from neighbouring sites
*wR*(*F*^2^) = 0.102	H-atom parameters constrained
*S* = 1.17	*w* = 1/[σ^2^(*F*_o_^2^) + (0.0287*P*)^2^ + 2.7247*P*] where *P* = (*F*_o_^2^ + 2*F*_c_^2^)/3
2493 reflections	(Δ/σ)_max_ < 0.001
182 parameters	Δρ_max_ = 0.98 e Å^−^^3^
0 restraints	Δρ_min_ = −0.31 e Å^−^^3^

### Special details

**Table:**

Geometry. All e.s.d.'s (except the e.s.d. in the dihedral angle between two l.s. planes) are estimated using the full covariance matrix. The cell e.s.d.'s are taken into account individually in the estimation of e.s.d.'s in distances, angles and torsion angles; correlations between e.s.d.'s in cell parameters are only used when they are defined by crystal symmetry. An approximate (isotropic) treatment of cell e.s.d.'s is used for estimating e.s.d.'s involving l.s. planes.
Refinement. Refinement of *F*^2^ against ALL reflections. The weighted *R*-factor *wR* and goodness of fit *S* are based on *F*^2^, conventional *R*-factors *R* are based on *F*, with *F* set to zero for negative *F*^2^. The threshold expression of *F*^2^ > σ(*F*^2^) is used only for calculating *R*-factors(gt) *etc*. and is not relevant to the choice of reflections for refinement. *R*-factors based on *F*^2^ are statistically about twice as large as those based on *F*, and *R*- factors based on ALL data will be even larger.

### Fractional atomic coordinates and isotropic or equivalent isotropic displacement parameters (Å^2^)

**Table:**

	*x*	*y*	*z*	*U*_iso_*/*U*_eq_	
S1	0.64889 (9)	0.08386 (5)	−0.14263 (5)	0.01840 (19)	
Cl1	0.92712 (10)	0.58799 (5)	0.13883 (6)	0.0241 (2)	
Cl2	0.74727 (10)	0.62951 (5)	−0.09604 (6)	0.0243 (2)	
N1	0.7016 (3)	0.19511 (15)	0.01236 (18)	0.0156 (5)	
N2	0.2888 (3)	0.16439 (17)	0.1598 (2)	0.0228 (6)	
N3	0.1985 (3)	0.01685 (18)	−0.1358 (2)	0.0276 (6)	
C1	0.5784 (4)	0.13843 (17)	−0.0353 (2)	0.0146 (6)	
C2	0.8768 (4)	0.1872 (2)	−0.0228 (2)	0.0194 (6)	
H2A	0.9508	0.1414	0.0192	0.023*	
H2B	0.9410	0.2450	−0.0150	0.023*	
C3	0.8370 (4)	0.1601 (2)	−0.1382 (2)	0.0199 (6)	
H3A	0.9408	0.1297	−0.1607	0.024*	
H3B	0.8047	0.2127	−0.1843	0.024*	
C4	0.4095 (4)	0.11831 (18)	−0.0098 (2)	0.0161 (6)	
C5	0.3460 (4)	0.14437 (18)	0.0850 (2)	0.0174 (6)	
C6	0.2938 (4)	0.06174 (19)	−0.0798 (2)	0.0184 (6)	
C7	0.6790 (4)	0.26246 (19)	0.0927 (2)	0.0177 (6)	
H7A	0.5539	0.2575	0.1076	0.021*	
C8	0.8041 (4)	0.2423 (2)	0.1955 (2)	0.0242 (7)	
H8A	0.7885	0.1800	0.2160	0.036*	
H8B	0.7762	0.2821	0.2516	0.036*	
H8C	0.9279	0.2518	0.1850	0.036*	
C9	0.6999 (4)	0.35513 (18)	0.0458 (2)	0.0158 (6)	
C10	0.7943 (4)	0.42212 (19)	0.1048 (2)	0.0178 (6)	
H10A	0.8506	0.4103	0.1752	0.021*	
C11	0.8072 (4)	0.50688 (18)	0.0613 (2)	0.0173 (6)	
C12	0.7274 (4)	0.52438 (19)	−0.0414 (2)	0.0187 (6)	
C13	0.6305 (4)	0.45847 (19)	−0.1013 (2)	0.0197 (6)	
H13A	0.5743	0.4708	−0.1716	0.024*	
C14	0.6165 (4)	0.37428 (19)	−0.0575 (2)	0.0186 (6)	
H14A	0.5494	0.3291	−0.0981	0.022*	

### Atomic displacement parameters (Å^2^)

**Table:**

	*U*^11^	*U*^22^	*U*^33^	*U*^12^	*U*^13^	*U*^23^
S1	0.0204 (4)	0.0169 (4)	0.0180 (4)	0.0004 (3)	0.0032 (3)	−0.0022 (3)
Cl1	0.0280 (4)	0.0179 (4)	0.0271 (4)	−0.0050 (3)	0.0062 (3)	−0.0057 (3)
Cl2	0.0257 (4)	0.0176 (4)	0.0315 (4)	0.0026 (3)	0.0111 (3)	0.0080 (3)
N1	0.0142 (12)	0.0136 (12)	0.0192 (12)	−0.0005 (9)	0.0035 (9)	0.0004 (9)
N2	0.0222 (13)	0.0214 (13)	0.0257 (14)	−0.0027 (10)	0.0068 (11)	0.0004 (11)
N3	0.0236 (14)	0.0267 (15)	0.0314 (15)	−0.0036 (11)	0.0010 (12)	−0.0027 (12)
C1	0.0171 (14)	0.0111 (13)	0.0149 (13)	0.0047 (10)	0.0002 (11)	0.0030 (10)
C2	0.0134 (14)	0.0202 (15)	0.0250 (15)	−0.0009 (11)	0.0038 (11)	−0.0006 (12)
C3	0.0170 (14)	0.0221 (15)	0.0217 (15)	−0.0002 (12)	0.0063 (12)	0.0011 (12)
C4	0.0170 (14)	0.0119 (13)	0.0183 (14)	−0.0010 (11)	−0.0005 (11)	0.0021 (11)
C5	0.0141 (14)	0.0124 (14)	0.0248 (16)	0.0001 (10)	0.0001 (12)	0.0037 (12)
C6	0.0177 (14)	0.0183 (15)	0.0198 (15)	0.0008 (12)	0.0047 (12)	0.0037 (12)
C7	0.0170 (14)	0.0177 (15)	0.0193 (15)	−0.0024 (11)	0.0061 (11)	−0.0039 (11)
C8	0.0330 (17)	0.0201 (15)	0.0191 (15)	−0.0044 (13)	0.0032 (13)	0.0022 (12)
C9	0.0137 (13)	0.0135 (14)	0.0213 (15)	−0.0006 (10)	0.0058 (11)	−0.0005 (11)
C10	0.0168 (14)	0.0203 (15)	0.0174 (14)	0.0020 (11)	0.0059 (11)	−0.0023 (11)
C11	0.0151 (14)	0.0128 (14)	0.0255 (15)	−0.0006 (11)	0.0074 (11)	−0.0044 (11)
C12	0.0186 (14)	0.0162 (14)	0.0232 (15)	0.0030 (11)	0.0095 (12)	0.0035 (12)
C13	0.0178 (14)	0.0222 (15)	0.0195 (15)	0.0039 (12)	0.0040 (11)	0.0016 (12)
C14	0.0149 (14)	0.0186 (15)	0.0219 (15)	−0.0018 (11)	0.0019 (11)	−0.0032 (12)

### Geometric parameters (Å, °)

**Table:**

S1—C1	1.751 (3)	C4—C6	1.429 (4)
S1—C3	1.821 (3)	C7—C8	1.526 (4)
Cl1—C11	1.732 (3)	C7—C9	1.528 (4)
Cl2—C12	1.736 (3)	C7—H7A	1.0000
N1—C1	1.336 (3)	C8—H8A	0.9800
N1—C7	1.468 (3)	C8—H8B	0.9800
N1—C2	1.474 (4)	C8—H8C	0.9800
N2—C5	1.150 (4)	C9—C10	1.384 (4)
N3—C6	1.151 (4)	C9—C14	1.402 (4)
C1—C4	1.404 (4)	C10—C11	1.394 (4)
C2—C3	1.514 (4)	C10—H10A	0.9500
C2—H2A	0.9900	C11—C12	1.382 (4)
C2—H2B	0.9900	C12—C13	1.386 (4)
C3—H3A	0.9900	C13—C14	1.389 (4)
C3—H3B	0.9900	C13—H13A	0.9500
C4—C5	1.427 (4)	C14—H14A	0.9500
			
C1—S1—C3	91.05 (13)	N1—C7—H7A	107.6
C1—N1—C7	127.2 (2)	C8—C7—H7A	107.6
C1—N1—C2	114.2 (2)	C9—C7—H7A	107.6
C7—N1—C2	118.6 (2)	C7—C8—H8A	109.5
N1—C1—C4	129.0 (3)	C7—C8—H8B	109.5
N1—C1—S1	112.0 (2)	H8A—C8—H8B	109.5
C4—C1—S1	119.0 (2)	C7—C8—H8C	109.5
N1—C2—C3	105.5 (2)	H8A—C8—H8C	109.5
N1—C2—H2A	110.6	H8B—C8—H8C	109.5
C3—C2—H2A	110.6	C10—C9—C14	118.9 (3)
N1—C2—H2B	110.6	C10—C9—C7	121.3 (2)
C3—C2—H2B	110.6	C14—C9—C7	119.7 (2)
H2A—C2—H2B	108.8	C9—C10—C11	120.3 (3)
C2—C3—S1	103.50 (19)	C9—C10—H10A	119.9
C2—C3—H3A	111.1	C11—C10—H10A	119.9
S1—C3—H3A	111.1	C12—C11—C10	120.2 (3)
C2—C3—H3B	111.1	C12—C11—Cl1	121.6 (2)
S1—C3—H3B	111.1	C10—C11—Cl1	118.2 (2)
H3A—C3—H3B	109.0	C11—C12—C13	120.3 (3)
C1—C4—C5	125.5 (2)	C11—C12—Cl2	120.0 (2)
C1—C4—C6	118.4 (3)	C13—C12—Cl2	119.6 (2)
C5—C4—C6	115.9 (2)	C12—C13—C14	119.3 (3)
N2—C5—C4	177.5 (3)	C12—C13—H13A	120.3
N3—C6—C4	179.0 (3)	C14—C13—H13A	120.3
N1—C7—C8	110.0 (2)	C13—C14—C9	120.9 (3)
N1—C7—C9	108.5 (2)	C13—C14—H14A	119.6
C8—C7—C9	115.4 (2)	C9—C14—H14A	119.6
			
C7—N1—C1—C4	−11.5 (4)	C1—N1—C7—C9	−114.5 (3)
C2—N1—C1—C4	169.1 (3)	C2—N1—C7—C9	64.9 (3)
C7—N1—C1—S1	169.1 (2)	N1—C7—C9—C10	−138.4 (3)
C2—N1—C1—S1	−10.3 (3)	C8—C7—C9—C10	−14.5 (4)
C3—S1—C1—N1	−11.7 (2)	N1—C7—C9—C14	44.2 (3)
C3—S1—C1—C4	168.8 (2)	C8—C7—C9—C14	168.0 (3)
C1—N1—C2—C3	32.4 (3)	C14—C9—C10—C11	−0.6 (4)
C7—N1—C2—C3	−147.1 (2)	C7—C9—C10—C11	−178.1 (2)
N1—C2—C3—S1	−37.5 (2)	C9—C10—C11—C12	−0.7 (4)
C1—S1—C3—C2	28.5 (2)	C9—C10—C11—Cl1	−180.0 (2)
N1—C1—C4—C5	−11.2 (5)	C10—C11—C12—C13	1.5 (4)
S1—C1—C4—C5	168.2 (2)	Cl1—C11—C12—C13	−179.3 (2)
N1—C1—C4—C6	173.6 (3)	C10—C11—C12—Cl2	−178.8 (2)
S1—C1—C4—C6	−7.0 (3)	Cl1—C11—C12—Cl2	0.4 (3)
C1—C4—C5—N2	169 (7)	C11—C12—C13—C14	−0.9 (4)
C6—C4—C5—N2	−16 (7)	Cl2—C12—C13—C14	179.4 (2)
C1—C4—C6—N3	−147 (20)	C12—C13—C14—C9	−0.5 (4)
C5—C4—C6—N3	37 (20)	C10—C9—C14—C13	1.2 (4)
C1—N1—C7—C8	118.5 (3)	C7—C9—C14—C13	178.8 (3)
C2—N1—C7—C8	−62.1 (3)		

### Hydrogen-bond geometry (Å, °)

**Table:**

*D*—H···*A*	*D*—H	H···*A*	*D*···*A*	*D*—H···*A*
C3—H3A···N3^i^	0.99	2.57	3.477 (4)	153
C7—H7A···Cl2^ii^	1.00	2.83	3.623 (3)	137

Symmetry codes: (i) *x*+1, *y*, *z*; (ii) −*x*+1, −*y*+1, −*z*.
